# The −44 C/G (rs1800972) polymorphism of the β‐defensin 1 is associated with increased risk of developing type 2 diabetes mellitus

**DOI:** 10.1002/mgg3.509

**Published:** 2018-12-13

**Authors:** Marco Antonio Martinez‐Rios, Gilberto Vargas‐Alarcon, Marco Antonio Peña‐Duque, Oscar Perez‐Mendez, Jose Manuel Rodriguez‐Perez, Nonanzit Perez‐Hernandez, Gabriel Herrera‐Maya, Rosalinda Posadas‐Sanchez, Carlos Posadas‐Romero, Jose Manuel Fragoso

**Affiliations:** ^1^ Interventional Cardiology Instituto Nacional de Cardiología Ignacio Chávez Mexico City Mexico; ^2^ Department of Molecular Biology Instituto Nacional de Cardiología Ignacio Chávez Mexico City Mexico; ^3^ Department of Endocrinology Instituto Nacional de Cardiología Ignacio Chávez Mexico City Mexico

**Keywords:** diabetes, genomics, susceptibility, β‐defensin 1

## Abstract

**Background:**

The aim of this study was to establish the association of two polymorphisms of the β‐defensin 1 gene (*DEFB1,* OMIM#602056*)* with the risk of developing type 2 diabetes mellitus (T2DM) in a group of Mexican patients.

**Methods:**

The 5′UTR −20 G/A, and −44 C/G polymorphisms of *DEFB1* gene were genotyped by 5′ exonuclease TaqMan assays in a group of 252 patients with T2DM and 522 healthy control.

**Results:**

Under dominant and additive models adjusted for the risk factors, the C allele of the −44 C/G polymorphism was associated with increased risk of T2DM (OR = 1.63, 95% CI = 1.07–2.48, pC_dom_ = 0.021 and OR = 1.42, 95% CI = 1.05–1.91, pC_add_ = 0.023, respectively). In addition, the linkage disequilibrium analysis showed that AC haplotype was associated with an increased risk of developing T2DM (OR = 4.39, *p* = 0.04). The in‐silico analysis showed that the −44 C allele produces a binding site for the transcription factor *Ikaros* (*IK)*.

**Conclusion:**

This study demonstrates that the C allele of −44 C/G polymorphism, as well as haplotype AC are associated with the presence of T2DM in the Mexican population. The variation in this polymorphism of the *DEFB1* gene could increase the migration of the macrophages to pancreatic islets accelerate the β‐cell dysfunction in T2DM.

## 1 INTRODUCTION

Chronic low‐grade inflammation and activation of the innate immune system are associated with insulin resistance, and β‐cell dysfunction in type 2 diabetes mellitus (T2DM) (Ehses, Perren, Eppler, Ribaux, & Pospisilik, 2007; Pickup, 2004). Recent studies have reported that the infiltration of the macrophages to pancreatic islets accelerates the β‐cell dysfunction. These macrophages secrete chemokines and stimulate the immune cell migration, as well as release of pro‐inflammatory cytokines. In addition, the elevated glucose and palmitate concentrations increase chemokines release that induce granulocyte colony‐stimulating factor and macrophage inflammatory protein‐1 from human and mouse pancreatic islets both in vitro and in vivo (Ehses et al., 2007; Inoue et al., 2018).

Experimental studies have shown that the β‐defensin 1 plays an important role in activation of the innate immune system. This molecule acts as a chemoattractant of T lymphocytes, immature dendritic cells, monocytes, macrophages, and mast cells by interaction with CCR6, as well as in the release of pro‐inflammatory cytokines (Soruri, Grigat, Forssman, Riggert, & Zwirner, 2007; Yang et al., 1999). This molecule is expressed by epithelial cells of the skin, gut, respiratory and urogenital tissue, the pancreas and the kidney (Schnapp, Reid, & Harris, 1998; Zhao, Wang, & Lehrer, 1996). Nonetheless, as far we know, in the literature there is no evidence about the genetic basis and the function of β‐defensin 1 in the pancreas, as well as in the pathophysiology of the T2DM. However, recent reports have showed that the β‐defensin 1 protein expression, as well as the mRNA levels increased in HMC and HEK‐293 cells cultured with high levels of glucose and insulin (Barnea, Madar, & Froy, 2008; Malik & Al‐Kafaji, 2007).

The β‐defensin 1 is encoded by *DEFB1* gene (OMIM#602056) that is located on the chromosome 8p22‐p23 region (Hollox, Barber, Brookes, & Armour, 2008). Recent studies have associated two single‐nucleotide polymorphisms (−20 G/A [rs11362, NM_005218.3:c.−20G>A] and −44 C/G [rs1800972, NM_0055218.3:c.−44C>G]) located in 5'UTR region of the gene with the expression and function of the β‐defensin 1 (Sun et al., 2006). In addition, these polymorphisms have been associated with the developing of inflammatory diseases as lepromatous leprosy, systemic lupus erythematosus, and recently with T2DM (Nemeth et al., 2014; Prado‐Montes de Oca, 2009; Sandrin‐Garcia et al., 2012).

According with the antecedents and considering that the T2DM affect more than 550 million people all over the globe (American Diabetes Association, 2013; Chen, Magliano, & Zimmet, 2011), the aim of this study was to establish the association of two polymorphisms (−20 G/A [rs11362] and −44 C/G [rs1800972]) of the *DEFB1* gene with the risk of developing T2DM in a group of Mexican patients.

## 2 MATERIALS AND METHODS

### 2.1 Population study

The study included 774 Mexican Mestizos, 252 consecutive patients with T2DM (30–78 years old) and 552 healthy controls (26–78 years old). From July 2010 to July 2015, 252 patients (189 men and 63 women, with a mean age of 58.5 ± 10.7) were referred to the Instituto Nacional de Cardiologia Ignacio Chavez. The diagnosis of T2DM was made according to the American Diabetes Association (ADA) and American Heart Association (AHA) based on three criteria, (a) an HbA1c value ≥6.5%, (b) fasting plasma glucose levels ≥126 mg/dl, (c) 2‐hr plasma glucose ≥200 mg/dl during an oral glucose tolerance test. The test was performed as described by the World Health Organization, using a glucose load containing the equivalent of 75 g anhydrous glucose dissolved in water (American Diabetes Association, 2013; Fox et al., 2015). Comorbidities such as BMI (weight [kg]/[height [m]^2^), gender, age, hypertension, dyslipidemia, smoking, and alcohol habit were recorded. Blood pressure was measured three times by trained personnel using sphygmomanometry. Hypertension was defined as systolic blood pressure ≥140 mmHg and/or diastolic blood pressure ≥90 mmHg or the use of oral antihypertensive therapy. Plasma lipid concentrations were determined after a 12‐hr overnight fast, EDTA blood samples were drawn and centrifuged within 15 minutes after collection; plasma was separated into aliquots and were analyzed within 24‐hr after blood sample collection. Cholesterol and triglycerides plasma concentrations were determined by enzymatic/colorimetric assays (Randox Laboratories, UK). The phosphotungstic acid‐Mg2+ method was used to determine HDL‐C concentrations. LDL‐cholesterol (LDL‐C) was estimated in samples with a triglyceride level lower than 400 mg/dl, using the Friedewald formula, modified by DeLong (DeLong, DeLong, Wood, & Lippel, 1986). We followed the National Cholesterol Education Project (NCEP) Adult Treatment Panel (ATP III) guidelines to defined dyslipidemia with the following levels: cholesterol > 200 mg/dl, LDL‐C > 130 mg/dl, HDL‐C < 40 mg/dl, and triglyceride > 150 mg/dl (https://www.nhlbi.nih.gov/guidelines/cholesterol/atp3_rpt.htm). Individuals with type 1 diabetes (T1D), maturity‐onset diabetes of the young (MODY), secondary diabetes due to hemochromatosis, cystic fibrosis, or pancreatitis or individuals with a family member with T1D were excluded from the study. The 522 healthy individuals (395 men and 63 women, with a mean age 53.6 ± 7.52) belong from the Genetics of Atherosclerosis Disease (GEA) Mexican study. All subjects were asymptomatic and apparently healthy individuals without a family history of premature CAD, atherosclerosis, and T2DM who were recruited during June 2009 to June 2013 among blood bank donors and with the assistance of brochures posted in social service centers. The exclusion criteria included the use of anti‐dyslipidemic or anti‐inflammatory drugs at the time of the study, congestive heart failure, and liver, renal, thyroid, or oncological disease (Posadas‐Sanchez et al., 2017). In addition, control subjects included in this study had a coronary calcium score = 0 as determined by computed tomography, indicating the absence of subclinical atherosclerosis in these individuals. All subjects included in this study were ethnically matched and considered Mexican mestizos if they had been born in Mexico and if their family had lived in the country for at least three generations. A Mexican mestizo is defined as someone born in Mexico and a descendant of both the original autochthonous inhabitants of the region and of individuals, mainly Spaniards of Caucasian and/or Black origin, who came to America during the sixteenth century. The study complies with the declaration of Helsinki and was approved by the Ethics Committee of the Instituto Nacional de Cardiología Ignacio Chávez (INCICH). All participants provided written informed consent.

### 2.2 Genetic analysis

The GenBank reference for *DEFB1* is NC_000008.11. DNA extraction was performed from white blood cells as previously described (Lahiri & Nurnberger, 1991). The genotyping of the −20 G/A (rs11362, NM_005218.3:c.−20G>A) and −44 C/G (rs1800972, NM_0055218.3:c.−44C>G) polymorphisms was made using 5′ exonuclease TaqMan assays as indicated by the manufacturer (Applied Biosystems, Foster City, USA). Samples previously sequenced were included as positive controls

### 2.3 Functional prediction analysis

Analysis in silico was made with the SNP Function Prediction (https://snpinfo.niehs.nih.gov/cgi-bin/snpinfo/snpfunc.cgi) program. This program analyze the location of the SNP (e.g., 5′‐upstream, 3′‐untranslated regions, intronic) and its possible functional effects, such as amino acid changes in protein structure, transcription factor binding sites in promoter or intronic enhancer regions, and alternative splicing regulation by disrupting exonic splicing enhancers or silencers (Xu & Taylor, 2009).

### 2.4 Statistical analysis

Considering that the continuous variables such as age, body mass index, blood pressure, glucose, total cholesterol, HDL‐C, LDL‐C, and triglycerides did not have normal behavior, they were analyzed using the Mann–Whitney *U* test. On the other hand, the categorical variables such as gender, alcohol consumption, and smoking habit were analyzed with the chi‐square test or Fisher’s exact test. The association of the polymorphisms with T2DM was analyzed by logistic regression using the following models: co‐dominant, dominant, recessive, heterozygous, and additive. Models were constructed in order to identify the variables that better explain the risk of developing T2DM between the study groups. Furthermore, models were built which incorporated one variable at a time, whereas final models included variables with biological relevance, statistical significance or both. Confounding bias was accepted when changes in estimated odds ratios (ORs) were equal or >10%. When a principal effect model was reached, the effect modification was also tested and interaction terms were constructed between the polymorphisms and various variables; the terms were included in the model when the significance of the *p*‐value was higher or equal to 0.05. All *p*‐values were corrected (pC) by the Bonferroni test. *p*C values <0.05 were considered statistically significant, and all odds ratios (OR) are presented with 95% confidence intervals. The occurrence of the T2DM in our population was based in the OR values: OR = 1 does not affect odds of developing T2DM, OR > 1 is associated with higher odds of developing T2DM, and OR < 1 is associated with lower odds of developing T2DM. The linkage disequilibrium analysis (LD, D") of the polymorphisms as well as the haplotype construction was performed with Haploview version 4.1 (Broad Institute of Massachusetts Institute of Technology and Harvard University, Cambridge, MA, USA). The analysis of data was performed with SPSS version 18.0 (SPSS, Chicago, IL) statistical package. The statistical power to detect association of the polymorphisms with T2DM was 0.80 and was estimated with QUANTO software (https://biostats.usc.edu/software).

## 3 RESULTS

### 3.1 Characteristics of the study population

Baseline characteristics of the T2DM patients and healthy controls included in the study are presented in Table 1.

**Table 1:** Demographic characteristics and biochemical parameters of the studied individuals

	T2DM (*n* = 252)	Healthy controls (*n* = 522)	*p‐*Value
	Median (percentile 25–75)	Median (percentile 25–75)	
Age (years)	58 (52–65)	53 (48–58)	<0.001
BMI (kg/m^2^)	26.5 (25–29)	28 (25–30)	0.026
Blood pressure (mmHg)			
Systolic	130 (112–150)	115 (107–126)	<0.001
Diastolic	80 (70–90)	72 (66–77.5)	<0.001
Total cholesterol (mg/dl)	165 (128–196)	189 (166–210)	<0.001
HDL‐C (mg/dl)	37 (32–46)	43 (35–53)	<0.001
LDL‐C (mg/dl)	100 (71–132)	115 (96–134)	0.001
Triglycerides (mg/dl)	150 (109–201)	149 (111–209)	0.958
Gender *n* (%)			
Male	189 (75)	395 (76)	0.453
Female	63 (25)	127 (25)	
Smoking *n* (%)			
Yes	69 (27)	119 (23)	0.097
Alcohol *n* (%)			
Yes	50 (20)	376 (72)	<0.001
Hypertension (mmHg)			
Yes	149 (59)	194 (37)	<0.001
Dyslipidemia			
Yes	217 (86)	212 (40)	<0.001

Notes

T2DM, Type 2 diabetes mellitus.

Data are expressed as median and percentiles (25th‐75th). *p‐*values were estimated using Mann–Whitney *U* test continuous variables and chi‐square test for categorical values.

### 3.2 Allele and genotype frequencies

The observed and expected frequencies in the two polymorphic sites of the *DEFB1* gene were in Hardy–Weinberg equilibrium. The 5′UTR −20 G/A (rs11362) polymorphism frequency was similar in patients with T2DM and healthy controls. However, under dominant and additive models adjusted for gender, age, BMI, hypertension, dyslipidemia, and alcohol consumption, the *C* allele of the 5’UTR −44 C/G (rs1800972) polymorphism was associated with increased risk of developing T2DM (OR = 1.63, 95% CI = 1.07–2.48, pC_dom_ = 0.021 and OR = 1.42, 95% CI = 1.05–1.91, pC_add_ = 0.023, respectively) (Table 2).

**Table 2:** Association of the *DEFB1* SNPs with T2DM

*n* (genotype frequency)				MAF	Model	OR (95% CI)	*pC*
*DEFB1 5′UTR −20 G/A*							
rs11362							
Control (*n* = 522)	*GG*	*GA*	*AA*	*A*			
	205 (0.393)	242 (0.464)	75 (0.144)	0.375	Co‐dominant	0.78 (0.42–1.47)	0.38
					Dominant	0.75 (0.50–1.13)	0.17
					Recessive	0.92 (0.51–1.65)	0.78
T2DM (*n* = 252)	111 (0.440)	106 (0.421)	35 (0.139)	0.349	Heterozygous	0.78 (0.52–1.18)	0.24
					Additive	0.84 (0.63–1.13)	0.26
*DEFB1 5′UTR −44 C/G*							
rs1800972							
Control (*n* = 522)	*GG*	*GC*	*CC*	*C*			
	223 (0.427)	230 (0.441)	69 (0.132)	0.352	Co‐dominant	1.85 (0.97–3.54)	0.06
					Dominant	1.63 (1.07–2.48)	**0.021**
					Recessive	1.44 (0.79–2.61)	0.24
T2DM (*n* = 252)	90 (0.357)	126 (0.500)	36 (0.143)	0.393	Heterozygous	1.35 (0.90–2.03)	0.14
					Additive	1.42(1.05–1.91)	**0.023**

CI, confidence interval; MAF, minor allele frequency; OR, odds ratio; *pC, p*‐corrected; T2DM, type 2 diabetes mellitus;

The *p‐values* were calculated by logistic regression analysis and the ORs were adjusted for age, blood pressure, body mass index (BMI), total cholesterol, HDL‐C, LDL‐C, hypertension, dyslipidemia, smoking, and alcohol consumption. Bold numbers indicate significant associations. The GenBank reference of *DEFB1*: NC_000008.11.

### 3.3 Linkage disequilibrium analysis

We analyzed haplotypes using the Haploview version 4.1 program. In this analysis, the two SNPs (*DEFB1* 5′UTR −20 G/A [rs11362] and *DEFB1* 5′UTR −44 C/G [rs1800972]) showed strong linkage disequilibrium (D′ > 0.94) and four haplotypes were constructed: H1 (AG), H2 (GC), H3 (GG), and H4 (AC). Haplotype H4 was associated with an increased risk of developing T2DM as compared to controls (OR = 4.39, *p* = 0.04), whereas the distribution of the haplotypes H1, H2, and H3 was similar in T2DM patients and healthy controls (Table 3).

**Table 3:** Frequencies of haplotypes of the *DEFB1* gene (5′UTR −20 G/A and 5′UTR −44C/G) in T2DM patients and healthy controls

	T2DM (*n* = 252)	Controls (*n* = 522)	OR	95%CI	*p‐Value*
Haplotype	Hf	Hf			
*H1 (AG)*	0.332	0.371	0.84	0.67–1.05	0.13
*H2 (GC)*	0.376	0.348	1.18	0.92–1.51	0.20
H3 *(GG)*	0.274	0.275	1.09	0.83–1.43	0.53
H4 *(AC)*	0.016	0.004	4.39	1.07–18.00	**0.04**

Hf, Haplotype frequency; *p*,* p value;* T2DM, type 2 diabetes mellitus.

The order of the polymorphisms in the haplotypes is according to the positions in the chromosome (rs11362 and rs1800972). Bold numbers indicate significant associations. The GenBank reference of *DEFB1*: NC_000008.11.

### 3.4 Functional prediction

The functional prediction analysis showed that the presence of the *A* allele of the *DEFB1* 5′UTR −20 G/A (rs11362) polymorphism produces a binding motif for the breast cancer transcription factor *(BRCA)*. The analysis also revealed that the *C* allele of the *DEFB1* 5′UTR −44 C/G (rs1800972) polymorphism generates binding motifs for ikaros transcription factor (*IK*).

## 4 DISCUSSION

In the present work, two polymorphisms (−20 G/A and −44 C/G) located in the 5′ untranslated region (UTR) of *DEFB1* gene were analyzed in T2DM patients and healthy controls. The distribution of 5′UTR −20 G/A (rs11362) was similar in both groups. Nonetheless, the analysis of the 5′UTR −44 C/G (rs1800972) polymorphism showed that the C allele was associated with an increased risk of developing T2DM under dominant and additive model. To the best of our knowledge, this is one of the few studies that describe the association of these polymorphisms with T2DM. Agreement with our results, Nemeth et al., report that the −44 CC genotype of the 5′UTR −44 C/G polymorphism was associated with increased risk for developing diabetes (OR = 2.05) in a Hungarian population. In addition, the authors reported that the frequency of the −44 GG genotype was lower in patients with diabetes (2%) than in the healthy controls (9%), suggesting that this genotype has a protective role in the development of diabetes (Nemeth et al., 2014). The association of 5′UTR −44 C/G polymorphism with other inflammatory diseases support our findings. For example, Prado‐Montes de Oca et al., studied three SNPs (−20 G/A, −44 C/G, and −52 G/A) in 75 leprosy patients and 151 controls. In this study, the authors reported that the −44 C allele and “CGA” haplotype increased the risk of developing lepromatous leprosy (OR = 3.06 and OR = 2.25, respectively) in a population of northern Mexico (Prado‐Montes de Oca, 2009). Moreover, Sandrine‐Garcia et al., studied the same three SNPs in 139 systemic lupus erythematosus patients and 288 healthy controls. In this work, the authors reported that the −44 GC genotype was associated with increased risk of developing this disease (OR = 1.60) in a Brazilian population (Sandrin‐Garcia et al., 2012). Our findings are consistent with those studies that report positive associations of the −44 C/G polymorphism with some inflammatory diseases. Additionally, we observed strong linkage disequilibrium (D′ > 0.94) between the two studied polymorphisms (−20 G/A and −44 C/G), and one haplotype (AC) was associated with increased risk of developing T2DM (OR = 4.39, *p = *0.04). As can be seen, this haplotype bears the C allele, that was associated with the disease in the independent analysis of the polymorphisms. This result corroborates the participation of this allele as a risk factor in the developing of the T2DM. Nonetheless, we considered that our findings, as well as the reported by Nemeth et al. (2014), need to be corroborated in additional studies with a larger number of individuals in different populations. This could help to further define the true role of this polymorphism as a marker of risk for developing T2DM.

Moreover, we explored in silico the functional effect of the *DEFB1* 5′UTR‐20 G/A and *DEFB1* 5′UTR‐44 C/G polymorphisms. Although the 5′UTR‐20 G/A SNP was not associated with T2DM, the analysis in silico showed that the* A* allele produces a binding motif for the *BRCA1* transcription factor. The *BRCA1* tumor suppressor is a protein that has an important role in multiple biochemical and biological functions such as DNA repair, replication, cell cycle checkpoints, protein ubiquitination, transcriptional regulation, chromatin remodeling, and apoptosis (Takoaka & Miki, 2018). Recently, Kang et al. (2012) reported that *BRCA1* regulates the expression of insulin‐like growth factor receptor‐1 (IGF‐IR) in human breast cancer cell line (MCF7). In line with this data, Shukla et al. (2006) reported that the absence of *BRCA1* results in increased expression of IRS‐1 (insulin receptor substrate‐1) and increased levels of IGF‐1R in *BRCA1‐*deficient mice, and in cultured human cells. On the other hand, our in‐silico analysis showed that the −44 C allele produces a binding site for the *Ikaros* (*IK)* transcription factor; *IK* factor regulates the expression of several other transcription factors as *Cdx4* that participates in the activation of the *STAT4*, and in the regulation of the expression of several pro‐inflammatory cytokines (John & Ward, 2011). In addition, it has been recently showed that the *IK* factor down‐regulates to IGF‐IR, which has an important role as a risk factor for T2DM (Singleton & Feldman, 2001). Additional to this information, Sun et al. (2006) reported that the C allele of the 668 C/G (named also −44 C/G) polymorphism increased the transcriptional activity of β‐defensin 1 promoter in luciferase assays, suggesting that this allele has an important role in the overexpression of the β‐defensin 1.

Accordingly, with these data, we suggested that the carriers of the −44 C allele could increase binding of the *IK* factor, and in consequence, (a) affect the regulation the insulin‐like growth factor receptor (IGF‐IR), and (b) heighten expression of pro‐inflammatory cytokines. On the other hand, recent studies showed the β‐defensin 1 has an important role as chemoattractant molecule of macrophages through its interaction with CCR6 (Soruri et al., 2007; Yang et al., 1999). In this context, it could be speculated that the β‐defensin 1 may be involved in the increased infiltration of the macrophages to pancreatic islets, accelerating the β‐cell dysfunction. However, the functional consequence of this polymorphism deserves to be specifically addressed in future studies.

We recognize that our study has limitations as the limited size of the sample in the groups of study. The functional effect of the polymorphisms only was determined by informatics tools, so experimental designs are needed in order to corroborate this functional effect. In spite of these limitations, our study contributes to a new argument in which the 5′UTR −44 C/G polymorphism may have a role as a risk factor for T2DM.

In summary, this study demonstrates that the *C* allele of 5′ UTR −44 C/G polymorphism is associated with the presence of T2DM in the Mexican population. In addition, it was possible to distinguish one haplotype (AC) associated with the presence of T2DM. Nonetheless, due to the number of individuals included in our study and the specific genetic characteristics of the Mexican population, we considered that additional studies in a larger number of individuals and in other populations with different ethnic origins could help to define the true role of this polymorphism as susceptibility marker for developing T2DM and others inflammatory events.

## CONFLICT OF INTEREST

The authors declare that there are no competing interests regarding the publication of this article.

## AUTHORS CONTRIBUTION

Gilberto Vargas‐Alarcon and Marco Antonio Martinez‐Rios contributed equally to this study. Gilberto Vargas‐Alarcon and Jose Manuel Fragoso were responsible for the conception and design of the study. Jose Manuel Rodriguez‐Perez, Gabriel Herrera‐Maya, and Carlos Posadas‐Romero, participated in the generation and collection of the samples. Marco Antonio Martinez‐Ríos, Marco Antonio Peña‐Duque, and Nonanzit Perez‐Hernandez, contributed in the analysis, and interpretation of data. Drafting or revision of the manuscript was handled by Gilberto Vargas‐Alarcon, Oscar‐Perez‐Mendez and Jose Manuel Fragoso.

## References

[mgg3509-bib-0001] American Diabetes Association . (2013). Diagnosis and classification of diabetes mellitus. Diabetes Care, 36 (Suppl 1): S67–S74. 23264425 10.2337/dc13-S067 PMC3537273

[mgg3509-bib-0002] Barnea, M. , Madar, Z. , & Froy, O. (2008). Glucose and Insulin are needed for optimal defensin expression in human cell lines. Biochemical and Biophysical Research Communications, 367, 452–456. 10.1016/j.bbrc.2007.12.158 18178160

[mgg3509-bib-0003] Chen, L. , Magliano, D. J. , & Zimmet, P. Z. (2011). The worldwide epidemiology of type 2 diabetes mellitus present and future perspective. Nature Reviews Endocrinology, 8, 228–236. 10.1038/nrendo.2011.183 22064493

[mgg3509-bib-0004] DeLong, D. M. , DeLong, E. R. , Wood, P. D. , Lippel, K. , & Rifkind, B. M. (1986). A comparison of methods for the estimation of plasma low‐ and very low‐density lipoprotein cholesterol. The Lipid Research Clinics Prevalence Study. JAMA, 256, 2372–2377. 10.1001/jama.1986.03380170088024 3464768

[mgg3509-bib-0005] Ehses, J. A. , Perren, A. , Eppler, E. , Ribaux, P. , Pospisilik, J. A. , Maor‐Cahn, R. , … Donath, M. Y. (2007). Increased number of islet‐associated macrophages in type 2 diabetes. Diabetes, 56, 2356–2370. 10.2337/db06-1650 17579207

[mgg3509-bib-0006] Fox, C. S. , Golden, S. H. , Anderson, C. , Bray, G. A. , Burke, L. E. , de Boer, I. H. , … Vafiadis, D. K. (2015). Update on prevention of cardiovascular disease in adults with type 2 diabetes mellitus in light of recent evidence: A scientific statement from the American heart association and the American diabetes association. Diabetes Care, 38, 1777–1803. 10.2337/dci15-0012 26246459 PMC4876675

[mgg3509-bib-0007] Hollox, E. J. , Barber, J. C. , Brookes, A. J. , & Armour, J. A. Defensins and the dynamic genome: What we can learn from structural variation at human chromosome band 8p23.1. Genome Research 2008;18: 1686–1697. 10.1101/gr.080945.108 18974263

[mgg3509-bib-0008] Inoue, H. , Shirakawa, J. , Togashi, Y. , Tajima, K. , Okuyama, T. , Kyohara, M. , … Terauchi, Y. (2018). Signaling between pancreatic B cells and macrophages via S100 calcium‐binding protein A8 exacerbates B‐cell apoptosis and islet inflammation. Journal of Biological Chemistry, 293, 5934–5946. 29496993 10.1074/jbc.M117.809228 PMC5912464

[mgg3509-bib-0009] John, L. B. , & Ward, A. C. (2011). The Ikaros gene family: Transcriptional regulators of hematopoiesis and immunity. Molecular Immunology, 48, 1272–1278. 10.1016/j.molimm.2011.03.006 21477865

[mgg3509-bib-0010] Kang, H. J. , Yi, Y. W. , Kim, H. J. , Hong, Y. B. , Seong, Y. S. , & Bae, I. (2012). BRCA1 negatively regulates IGF‐1 expression through an estrogen‐responsive element‐like site. Cell Death Disease., 3, e336 10.1038/cddis.2012.78 22739988 PMC3388245

[mgg3509-bib-0011] Lahiri, D. K. , & Nurnberger, J. I., Jr. (1991). A rapid non‐enzymatic method for the preparation HMW DNA from blood for RFLP studies. Nucleic Acids Research, 19, 5444. 1681511 10.1093/nar/19.19.5444 PMC328920

[mgg3509-bib-0012] Malik, A. N. , & Al‐Kafaji, G. (2007). Glucose regulation of beta‐defensin‐1 mRNA in human renal cells. Biochemical and Biophysical Research Communications, 353, 318–323. 17187760 10.1016/j.bbrc.2006.12.037

[mgg3509-bib-0013] Nemeth, B. C. , Varkonyi, T. , Somogyvari, F. , Lengyel, C. , Fehertemplomi, K. , Nyiraty, S. , … Mandi , Y. (2014). Relevance of α‐defensins (HNP1‐3) and defensin B‐1 in diabetes. World Journal of Gastroenterology, 20, 9128–9137. 25083086 10.3748/wjg.v20.i27.9128 PMC4112898

[mgg3509-bib-0014] Pickup, J. C. (2004). Inflammation and activated innate immunity in the pathogenesis of type 2 diabetes. Diabetes Care, 27, 813–823. 10.2337/diacare.27.3.813 14988310

[mgg3509-bib-0015] Posadas‐Sanchez, R. , Perez‐Hernandez, N. , Angeles‐Martinez, J. , Lopez‐Bautista, F. , Villarreal‐Molina, T. , Rodríguez‐Perez, J. M. ,… Vargas‐Alarcón , G. (2017). Interleukin 35 polymorphisms are associated with decreased risk of premature coronary artery disease, metabolic parameters, and IL‐35 levels: The genetics of atherosclerotic disease (GEA) study. Mediators of Inflammation, 2017: 6012795. 28321150 10.1155/2017/6012795 PMC5340958

[mgg3509-bib-0016] Prado‐Montes de Oca, E. , Velarde‐Felix, J. S. , Rios‐Tostado, J. J. , Picos‐Cardenas, V. J. , & Figueroa, L. E. (2009). SNP 668C (‐44) alters a NF‐kB1 putative binding site in non‐coding strand of human Β‐defensin 1 (DEFB1) and is associated with lepromatous leprosy. Infection, Genetics and Evolution, 9, 617–625. 10.1016/j.meegid.2009.03.006 19460328

[mgg3509-bib-0017] Sandrin‐Garcia, P. , Brandao, L. A. C. , Guimaraes, R. L. , Pancoto, J. A. , Donadi, E. A. , Lima‐Fiho, J. L. , … Crovella, S. (2012). Fucntional single‐nucleotide polymorphism in the DEFB1 gene are associated with systemic lupus erytematosus in southern Barzilians. Lupus, 21, 625–631. 22323338 10.1177/0961203312436858

[mgg3509-bib-0018] Schnapp, D. , Reid, C. J. , & Harris, A. (1998). Localization of expression of human beta defensin‐1 in the pancreas and kidney. Journal of Pathology, 186, 99–103. 10.1002/(SICI)1096-9896(199809)186:1<99:AID-PATH133>3.0.CO;2-#; 9875146

[mgg3509-bib-0019] Shukla, V. , Coumoul, X. , Cao, L. , Wang, R. H. , Xiao, C. , Xu, X. , … Deng, C. (2006). Absence of the full‐length breast cancer‐associated gene‐1 leads to increased expression of insulin‐like growth factor signaling axis members. Cancer Research, 66, 7151–7157. 10.1158/0008-5472.CAN-05-4570 16849561

[mgg3509-bib-0020] Singleton, J. B. , & Feldman, E. L. (2001). Insulin‐like growth factor‐I in muscle metabolism and myotherapies. Neurobiology of Diseases, 8, 541–554. 10.1006/nbdi.2001.0416 11493020

[mgg3509-bib-0021] Soruri, A. , Grigat, J. , Forssman, U. , Riggert, J. , & Zwirner, J. (2007). B‐defensins chemoattract macrophages and mast cells but not lymphocytes and dendritic cells: CCR4 is not involved. European Journal of Immunology, 37, 2474–2486. 17705135 10.1002/eji.200737292

[mgg3509-bib-0022] Sun, C. Q. , Arnold, R. , Fernandez‐Golarz, C. , Parrish, A. B. , Almekinder, T. , He, J. , Ho, S. M. , … Petros, J. A. (2006). Human B‐defensin‐1 a potential chromosome 8p tumor suppressor. Control of transcription and induction of apoptosis in renal cell carcinoma. Cancer Research, 66, 8542–8549. 16951166 10.1158/0008-5472.CAN-06-0294

[mgg3509-bib-0023] Takoaka, M. , & Miki, Y. (2018). BRCA1 gene: Function and deficiency. International Journal of Clinical Oncology, 23, 36–44. 10.1007/s10147-017-1182-2 28884397

[mgg3509-bib-0024] Xu, Z. , & Taylor, J. A. SNPinfo: Integrating GWAS and candidate gene information into functional SNP selection for genetic association studies. Nucleic Acids Research 2009;37(Web Server Issue): W600–W605. 10.1093/nar/gkp290 19417063 PMC2703930

[mgg3509-bib-0025] Yang, D. , Chertov, O. , Bykovskaia, S. N. , Chen, Q. , Buffo, M. J. , Shogan, J. , … Oppenheim, J. J. (1999). Beta defensins: Linking innate and adaptive immunity through dendritic and T cell ccr6. Science, 286, 525–528. 10.1126/science.286.5439.525 10521347

[mgg3509-bib-0026] Zhao, C. , Wang, I. , & Lehrer, R. I. (1996). Widespread expression of betadefensin hBD‐1 in human secretory glands and epithelial cells. FEBS Letters, 396, 319–322. 10.1016/0014-5793(96)01123-4 8915011

